# A pilot trial of quetiapine, lithium, or placebo added to divalproex sodium for hypomanic or manic episodes in ambulatory adults with bipolar I disorder

**DOI:** 10.1186/s40345-022-00252-w

**Published:** 2022-03-02

**Authors:** Victoria E. Cosgrove, Santiago Allende, Iola Gwizdowski, E. Grace Fischer, Michael Ostacher, Trisha Suppes

**Affiliations:** 1 Bipolar and Depression Research Program, VA Palo Alto Health Care System, 3801 Miranda Avenue (151T), Palo Alto, CA 94304 USA; 2 Department of Psychiatry and Behavioral Sciences, Stanford University School of Medicine, 401 Quarry Road, Stanford, CA 94305 USA

**Keywords:** Monotherapy divalproex, Divalproex plus blinded lithium, Divalproex plus blinded quetiapine

## Abstract

**Background:**

Many patients with bipolar I disorder do not respond to monotherapy treatment with mood-stabilizing medications, and combination regimens are commonly used in both inpatient and outpatient settings for the acute and maintenance treatment of bipolar disorder. We studied whether combination therapy is more effective than monotherapy for the acute treatment of subjects with bipolar I disorder currently experiencing manic symptoms. The primary hypothesis was that combination treatments would be associated with greater reductions in symptoms of mania and hypomania than monotherapy alone. The secondary hypothesis was that combination therapies would be associated with lower depression levels than monotherapy alone. Last, a post-hoc exploratory aim was used to examine whether the effect of side effect severity on risk-of-dropout would be greater in combination therapies than in monotherapy alone.

**Results:**

In this 12-week, double-blind, placebo-controlled ambulatory pilot trial, participants (n = 75) with bipolar I disorder were randomly assigned to: (1) monotherapy divalproex plus placebo (DVP + PBO), (2) combination therapy of divalproex plus blinded lithium (DVP + Li) or (3) divalproex plus blinded quetiapine (DVP + QTP). Combination therapies (vs. monotherapy) were not associated with improved symptoms of mania, hypomania or depression. The effect of side effect severity on study retention did not differ between combination therapies and monotherapy. However, the risk-of-dropout was significantly greater in the DVP + Li arm versus the DVP + PBO arm.

**Conclusions:**

No longitudinal differences in mania, hypomania or depression were found between combination therapies and monotherapy. The effect of side effect severity on study retention did not differ between groups. Due to the small sample size and differential rates of attrition between treatment arms, results of this pilot trial must be interpreted with caution.

*Trial registration* ClinicalTrials.gov identifier: NCT00183443

## Background

Bipolar disorder is a chronic mental illness often requiring lifelong treatment (Suppes et al. 1991). Effectively treating bipolar disorder is often challenging, in part because of the disorder’s chronic and recurrent nature. Most treatment guidelines recommend monotherapy as a first-line treatment option, reserving treatment with multiple medications as an option for more severely ill or non-responsive patients. Yet, combination therapies are commonly used in both inpatient and outpatient settings as initial treatment (Yatham et al. 2018; Grande and Vieta 2015).

Adverse events are one major consequence of polypharmacy in bipolar I disorder, leading to diminished quality of life and global functioning, poorer medication adherence, and higher rates of treatment dropout (Brooks et al. 2010; Gaudiano et al. 2008; Scott 2002). Both patient nonadherence to psychotropic medication regimens and early dropout further complicate treatment, possibly explaining an efficacy-effectiveness gap observed in bipolar disorder research in which clinical trials report higher efficacy rates than those observed in clinical practice (Gaudiano et al. 2008; Scott 2002).

Patients with bipolar I disorder do not exclusively have episodes of mania or depression; they may also experience episodes of hypomania, and hypo/mania and depression with mixed features. In spite of this, the evidence base for the treatment of hypomanic episodes in bipolar I disorder is very limited, and to date there are no established treatments for hypomanic episodes. Little attention has been directed at these types of episodes as a treatment target, though it is recognized that hypomania itself can signal destabilization in patients with bipolar I disorder.

A growing body of evidence suggests negligible overall improved outcomes for combination therapies, indicating that the question of monotherapy versus combination therapy remains highly relevant (Nierenberg et al. 2013; Altshuler et al. 2017; Geddes et al. 2011). In clinical practice, divalproex is commonly combined with either quetiapine or lithium to treat hypomania or mania. The present pilot study is a 12-week acute phase, double-blind, placebo-controlled trial of monotherapy versus combination medication comparing open-label divalproex plus adjunctive blinded slow-release lithium carbonate, blinded quetiapine, or placebo for the treatment of hypomanic or manic symptoms during hypomanic, manic, or mixed episodes in outpatient participants with bipolar I disorder.

## Methods

This is a randomized, double-blind, placebo-controlled, 12-week pilot investigation of the safety and effectiveness of monotherapy divalproex plus placebo (DVP + PBO) versus combinations of divalproex plus blinded lithium (DVP + Li) or divalproex plus blinded quetiapine (DVP + QTP) for treatment of hypomania or mania in outpatients with bipolar I disorder.

### Procedures

This study was approved by Institutional Review Boards of the University of Texas Southwestern Medical Center and the Stanford University School of Medicine, written informed consent was obtained, and the study was registered at ClinicalTrials.gov (identifier: NCT00183443).

Outpatient participants diagnosed with bipolar I disorder as defined in DSM-IV between the ages of 18–65 and experiencing hypomania or mania with a Young Mania Rating Scale (YMRS) score of ≥ 15 were eligible for study entry (see Table 1 for inclusion/exclusion criteria). Although patients were excluded for current use of DVP + Li, patients were not excluded for current monotherapy use of DVP, Li or QTP without a history of intolerance, toxicity or adverse experiences. Once enrolled, participants were randomized to one of three treatment conditions: DVP + PBO (monotherapy), DVP + Li, or DVP + QTP. Participants were evaluated weekly for the first 4 weeks then at 2-week intervals until the completion of the 12-week study. Participants, study physicians, and raters were blind to group assignment.

**Table 1:** Inclusion and exclusion criteria

Inclusion criteria	Exclusion criteria
• English-speaking adults at least 18 years old • History of DSM-IV bipolar I disorder, confirmed by Structured Clinical Interview • Experiencing hypomania or mania with a score on Young Mania Rating Scale ≥ 15 and meeting DSM-IV criteria for a current hypomanic, manic, or mixed episode • Desire to seek treatment for bipolar I disorder • Written informed consent obtained and willingness to perform study procedures confirmed • Agree to taper existing ineffective medications and be randomized to one of the three study conditions • No use of antidepressants for 1 month prior to randomization (3 months for fluoxetine)	• Patients who are euthymic (well) on their current medications • Patients who are already taking combination DVP + Li or DVP + QTP • Patients with a history of partial or nonresponse to DVP, Li, QTP, or any combination of those medications, documented by serum levels • History of intolerance or toxicity to DVP, Li, or QTP • History of adverse experiences to DVP, Li, or QTP • Disorders that would contraindicate or limit the use of Li, including dermatological conditions, such as psoriasis or acne vulgaris, or kidney failure • Disorders that would contraindicate the use of DVP, including clinically significant active hepatitis or hepatic failure as evidenced by abnormal laboratory values • Impaired cardiac function as evidenced by positive EKG in subjects age 50 or over • Patients with unstable medical illnesses within the past 2 months • Current suicidal ideation or intent • Substance abuse or dependency within the past month • Pregnant (i.e., positive urine pregnancy test) or nursing women or planning to conceive

At screening, a diagnosis of bipolar I disorder (BDI) was confirmed with the Structured Clinical Interview for DSM-IV (SCID) (First et al. 1996). The screening visit also consisted of confirmation of inclusion and exclusion criteria, laboratory work, a physical exam including an eye exam, and an EKG (if subject was > 50 years old). Participants prescribed other psychiatric medications at study entry were simultaneously started on study medication and titrated off prior medications at a rate of 33% every 3–4 days to assure that the taper was complete by week 2 (Faedda et al. 1993). Randomization occurred if screening visit results confirmed eligibility. Efron’s biased coin design, with stratified randomization, separately for participants who reported rapid cycling in the preceding 12 months and those who did not, was used to ensure even distribution across treatment arms (Efron 1971).

At each study visit, study staff administered clinical assessment measures. The primary outcome measure was change in YMRS scores (Young et al. 1978), an 11-item clinician-rated measure providing data on degree and severity of manic symptoms. The Hamilton Rating Scale for Depression (HAM-D) (Hamilton 1960; Hamilton 1967; Williams 1988), a 17-item measure of symptom severity for depression, served as a secondary outcome measure. In addition to weight change, a total of 31 side effects were rated on a 4-point Likert-type scale ranging from 1 (*absent*) and 2 (*mild*) to 3 (*moderate*) and 4 (*severe*).

#### Blinded medication adjustments

A study psychiatrist blind to treatment assignment adjusted study medications based on clinical symptoms at each study visit. Open-label divalproex was titrated to a stable dose with periodic serum level checks on blood levels to confirm levels were within the therapeutic window (see below). Blinded medications were dosed at one capsule/day for the first 2 days, two capsules/day for the second 2 days, three capsules/day for the next 3 days, followed by four capsules/day for the remainder of the trial. Study medication was increased if a subject had no change or worsened, as evaluated by the CGI-BP and the YMRS. Same day oversight of all adjustments was by an unblinded psychiatrist to assure that doses and levels were in the prespecified range; the unblinded psychiatrist would substitute placebo if a dosage increase ordered by the blinded psychiatrist was outside the dosage range.

#### Dosing and serum levels of DVP

DVP was titrated as 250 mg/day × 2 days; 500 mg/day × 2 days; 750 mg/day until the next visit, at which time a serum blood level should be drawn. The approximate target dose range was 750–2000 mg/day, which can be administered in one dose. All subjects were maintained at a minimum level of DVP of 50 mg/L and a maximum of 125 mg/L. Patients unable to tolerate these minimums, despite decrease of blinded medication to minimum dosage/serum levels, were discontinued. Once this minimum of DVP was reached, all other medication adjustments took place only in the blinded medication (QTP, Li, or PBO).

#### Dosing and serum levels of lithium

The target goal for Li was 0.8 mEq/L or greater, and the minimum was 0.6 mEq/L. Lithium was started at 300 mg/day, and gradually increased to 900 mg/day over the course of a week (Table 3). Lithium level was drawn within 5 days after reaching minimum initial dosing of 900 mg/day (week 2), and at weeks 4, 6, 12, 20, and 26. Serum levels were targeted to 0.6 to < 1.2 mEg/L.

#### Dosing of quetiapine

QTP was initiated at 50 mg, increased to 100 mg, then by 100 mg/day until 400 mg was reached by Day 5 with a goal of 600 mg during week 2. In order to maintain with real world applicability, these targets were encouraged by an unblinded physician, but decreases or slower titration was allowed for tolerability. A minimum of 100 mg/day of QTP was required to remain in the study.

#### Discontinuation and use of other medications

Subjects were terminated from the protocol if the CGI-BP rating for change from the previous measurement was “much worse” or “very much worse” if confirmed at a return clinic visit. Subjects who were unable to maintain minimum doses or levels of any study medications (valproate serum level of 50 mg/mL, lithium level of 0.6 eEq/L, or quetiapine dose of 100 mg) due to side effects were discontinued from the study.

Short-term use of lorazepam up to 2 mg/day for a maximum of 5 consecutive days on no more than three occasions over the trial was permitted. No other adjunctive medication was allowed. For subjects who met response criteria at week 12 (YMRS < 8, HAM-D_17_ < 10), a 14-week continuation period was offered. Results from this continuation trial are not reported here.

### Statistical analysis

An a priori power analysis indicated that 35 participants per arm were required to obtain 80% power for detecting an effect size of 0.6, using a one-tailed test with an alpha level of 0.05. Due to dependency in the data, an intent-to-treat linear mixed modeling approach was used to examine the primary and secondary outcome measures, change in YMRS score and change in HAM-D score, respectively (Raudenbush and Bryk 2002). Linear mixed models use listwise deletion of individual observations rather than entire persons and maximum likelihood estimation to robustly handle data that are missing at random or completely at random (Snijders and Bosker 1999). Prior to all analyses, all continuous variables, with the exception of time, were centered at their arithmetic means.

A likelihood ratio test demonstrated that the best fitting model for mania and depression, fit separately, was a random linear time model. Both models were estimated using restricted maximum likelihood and included fixed effects for treatment condition (DVP + PBO vs. DVP + Li & DVP + PBO vs. DVP + QTP), time, their interaction and site. We did not include a 3-way interaction with site due to only six participants completing the trial at the Palo Alto VA. Separate linear mixed models were estimated to assess longitudinal differences in the monotherapy versus aggregated combination therapy groups (DVP + PBO vs. DVP + Li & DVP + QTP). A standardized coefficient for significant variables was derived by dividing the product of the coefficient and the standard deviation of the variable by the standard deviation of the outcome variable (Snijders and Bosker 1999). Diagnostic plots for level 1 and for level 2 did not reveal notable departures from normality, linearity, independence or constant variance.

Due to the small sample size and high rate of attrition in the present pilot study, in lieu of examining changes in the Clinical Global Impression Scale for Bipolar Disorder (CGI-BD), the Global Assessment of Functioning and the Social and Occupational Functioning Assessment Scale (SOFAS) as secondary outcome measures, a post-hoc exploratory analysis was used to investigate whether attrition differed among treatment arms as a function of side effect severity. In a Cox proportional hazards model, an interaction term between treatment group and side effect severity as a time-dependent covariate was used to examine whether the effect of treatment group on time-in-study depended on side effect severity. The side effect severity variable was derived by first recentering the scale to range from 0 to 3 (*absent* to *severe*) and then summing the side effect ratings for each participant. In a separate model, we tested an interaction between treatment group and number of side effects reported to compare with the side effect severity variable. Models were adjusted for YMRS and HAM-D scores.

A modified intention-to-treat analysis was performed, where only participants with at least one post-randomization follow-up visit were included in the Cox regression model. An examination of Schoenfeld residuals did not reveal violations of the proportional hazards assumption. Further, due to six of the participants completing the trial at the Palo Alto VA (vs. UT Southwestern Medical School), we estimated models with and without adjusting for the effect of site. Although a 3-way interaction would have been preferred, the unbalanced data would have rendered estimates less reliable. Last, separate models were estimated for monotherapy versus individual combination therapy (DVP + PBO vs. DVP + Li & DVP + PBO vs. DVP + QTP) and monotherapy versus aggregated combination therapy groups (DVP + PBO vs. DVP + Li & DVP + QTP).

All analyses were conducted with the R statistical computing language within the RStudio IDE (Winder et al. 2019; Team R 2020). Data cleaning and structuring were completed with the tidyverse set of packages, while the linear mixed models were estimated with the nlme package (Wickham 2017; Pinheiro et al. 2013). The sjPlot package was used to generate the linear mixed model table (Lüdecke 2018). The survival and survminer packages were used to estimate the survival functions (Therneau 2020; Kassambara 2018).

## Results

**Fig. 1:**
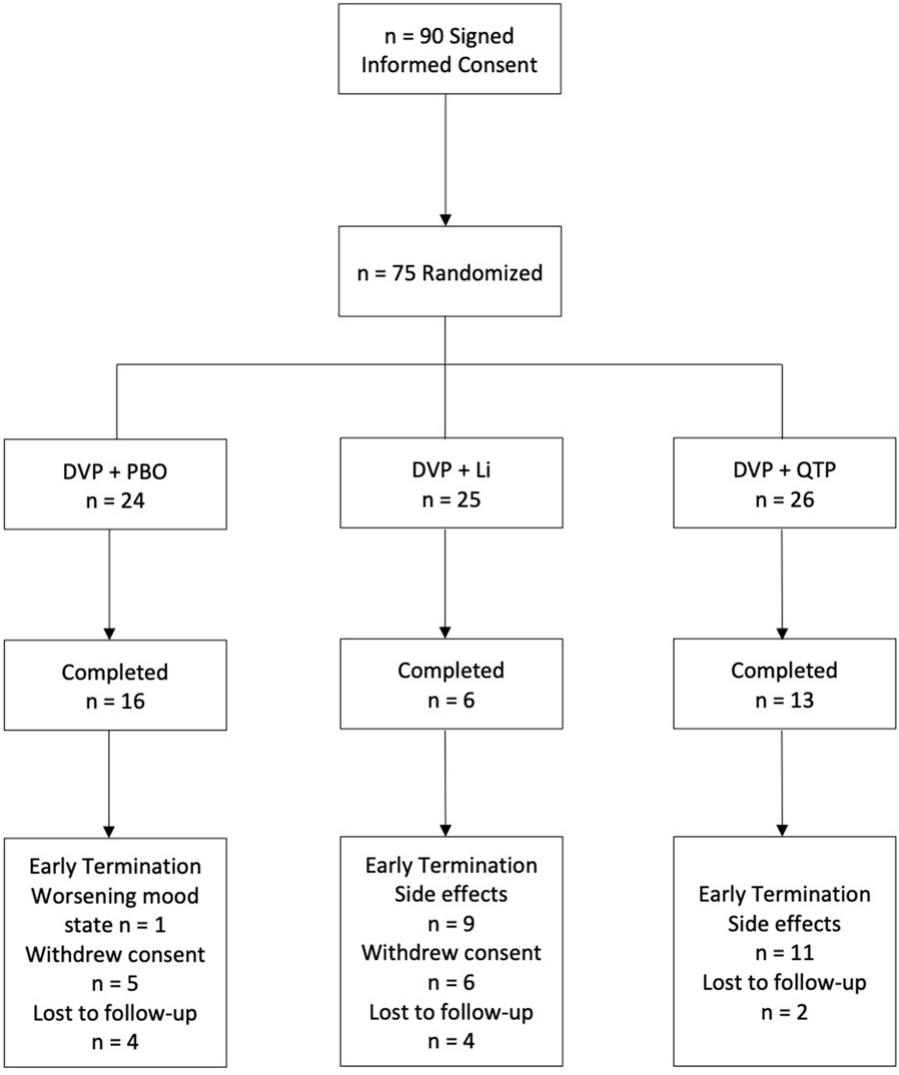
Consort diagram

Of the 75 participants who were included in the study (mean [*SD*] age = 35.5 [10.4] years; 50.7% female), 24 were randomized to the DVP + PBO arm (mean [*SD*] age = 38.8 [11.9] years; 41.7% female), 25 were randomized to DVP + Li (mean [*SD*] age = 33.3 [9.1] years; 48.0% female) and 26 were randomized to DVP + QTP (mean [*SD*] age = 34.5 [9.7] years; 61.5% female; see Fig. 1). As shown in Table 2, no differences in demographic or medical variables were found across the three groups. One subject was on divalproex at study start; otherwise all subjects were started on divalproex at baseline. The average number of reported side effects of at least moderate intensity in the entire cohort and per arm is shown in Table 3. Last, negligible differences in linear mixed model estimates were found between the individual and aggregated combination therapy models (see Table 4).

**Table 2:** Baseline characteristics of sample

	Entire cohort N = 75	DVP + PBO n = 24	DVP + Li n = 25	DVP + QTP n = 26	Significance test
Age at baseline (mean, SD)	35.5 (10.4)	38.8 (11.9)	33.3 (9.1)	34.5 (9.7)	F(2) = 1.91, p = 0.155
Women	38 (50.7%)	10 (41.7%)	12 (48.0%)	16 (61.5%)	χ^2^ (2) = 2.08, p = 0.354
Race/ethnicity					
Caucasian	53 (70.7%)	18 (75.0%)	17 (68.0%)	18 (69.2%)	χ^2^ (2) = 0.33, p = 0.848
Other	22 (29.3%)	6 (25.0%)	8 (32.0%)	8 (30.8%)	
YMRS score at baseline (mean, SD)	22.97 (6.20)	22.92 (5.72)	23.84 (7.49)	22.19 (5.32)	F(2) = 0.4449, p = 0.643
Mean HAM-D score at baseline (mean, SD)	13.57 (6.50)	13.88 (6.02)	13.20 (6.81)	13.65 (6.86)	F(2) = 0.0673, p = 0.935
Marital status					
Married or cohabitating	25 (33.3%)	7 (29.2%)	8 (32.0%)	10 (38.5%)	χ^2^ (2) = 0.52, p = 0.773
Single, divorced, widowed or separated	50 (66.7%)	17 (70.8%)	17 (68.0%)	16 (61.5%)	
Education					
High school diploma or less	19 (25.3%)	8 (33.3%)	5 (20.0%)	6 (23.1%)	χ^2^ (4) = 2.91, p = 0.573
Some college or vocational	33 (44.0%)	8 (33.3%)	11 (44.0%)	14 (53.8%)	
Associates degree or more	23 (30.7%)	8 (33.3%)	9 (36.0%)	6 (23.1%)	
Employment status					
Self, PT, or FT employment	47 (62.7%)	17 (70.8%)	14 (56.0%)	16 (61.5%)	χ^2^ (2) = 1.17, p = 0.556
Not employed	28 (37.3%)	7 (29.2%)	11 (44.0%)	10 (38.5%)	
Age at onset of first symptoms of depression	12.5 (6.9)	12.4 (8.0)	13.2 (6.4)	11.8 (6.3)	F(2) = 12.99, p = 0.763
Age at onset of first symptoms of mania	15.2 (12.6)	13.4 (10.3)	17.4 (18.4)	14.6 (5.6)	F(2) = 0.65, p = 0.524
Mean number of lifetime episodes of depression	3.5 (1.1)	3.4 (1.0)	3.7 (0.9)	3.5 (1.3)	F(2) = 0.50, p = 0.607
Mean number of lifetime episodes of mania	3.9 (0.6)	3.9 (0.7)	4 (0.0)	3.8 (0.9)	F(2) = 0.39, p = 0.679
Mean number of lifetime episodes of hypomania	4.0 (0.5)	4.1 (0.4)	3.9 (0.6)	4.0 (0.6)	F(2) = 1.33, p = 0.272
Age of diagnosis of any mood disorder	26.7 (11.2)	29.5 (11.2)	25.4 (10.2)	25.5 (11.9)	F(2) = 1.09, p = 0.342
Number of lifetime psychiatric hospitalizations	1.2 (3.7)	1.7 (6.1)	1.0 (1.7)	1.0 (1.8)	F(2) = 0.32, p = 0.725
Self-reported lifetime history of rapid cycling	60 (85.1%)	18 (75.0%)	23 (92.0%)	22 (88.0%)	χ^2^ (2) = 0.40, p = 0.818
Self-reported lifetime history of psychosis	26 (35.1%)	8 (33.3%)	10 (40%)	8 (32%)	χ^2^ (2) = 3.04, p = 0.219

*Significant at *p* < 0.05

**Table 3:** Reported side effects of at least moderate intensity judged to be possibly, probably, or definitely related to study medication

	Entire cohort N = 75	DVP + PBO n = 24	DVP + Li n = 25	DVP + QTP n = 26
Average number of side effects reported	5.4 (2.6)	6.5 (2.2)	4.5 (2.4)	5.4 (3.0)
Increased appetite	34 (45.3%)	11 (14.7%)	12 (16%)	11 (14.7%)
Sedation	32 (42.7%)	9 (12%)	2 (2.7%)	21 (28%)
Dry mouth	29 (38.7%)	6 (8%)	8 (10.7%)	15 (20%)
Nausea	25 (33.3%)	8 (10.7%)	9 (12%)	8 (10.7%)
Tiredness	23 (30.7%)	7 (9.3%)	4 (5.3%)	12 (16%)
Increased thirst	21 (28%)	2 (2.7%)	7 (9.3%)	12 (16%)
Upset stomach	21 (28%)	8 (10.7%)	7 (9.3%)	6 (8%)
Diarrhea	20 (26.7%)	9 (12%)	7 (9.3%)	4 (5.3%)
Gastrointestinal problems	18 (24%)	7 (9.3%)	8 (10.7%)	3 (4%)
Increased urinary frequency	16 (21.3%)	3 (4%)	9 (12%)	4 (5.3%)
Word finding difficulties	14 (18.7%)	1 (1.3%)	6 (8%)	7 (9.3%)
Slurred speech	10 (13.3%)	0 (0%)	3 (4%)	7 (9.3%)
Ataxia*	10 (13.3%)	0 (0%)	3 (4%)	7 (9.3%)
Weakness	9 (12%)	2 (2.7%)	2 (2.7%)	5 (6.7%)
Dizzy/lightheaded	9 (12%)	0 (0%)	2 (2.7%)	7 (9.3%)
Edema	9 (12%)	3 (4%)	2 (2.7%)	4 (5.3%)
Impaired memory	9 (12%)	1 (1.3%)	3 (4%)	5 (6.7%)
Feeling dull	9 (12%)	0 (0%)	2 (2.7%)	7 (9.3%)
Cognitive slowing	9 (12%)	0 (0%)	2 (2.7%)	7 (9.3%)
Joint/muscle aches	9 (12%)	4 (5.3%)	2 (2.7%)	3 (4%)
Tactile sensations	7 (9.3%)	1 (1.3%)	1 (1.3%)	5 (6.7%)
Increased appetite	34 (45.3%)	11 (14.7%)	12 (16%)	11 (14.7%)

*Significant at *p* < 0.05

**Table 4:** Longitudinal outcomes in YMRS and HAM-D: monotherapy versus aggregated combination therapies

Predictors	YMRS			HAM-D		
	Estimates	CI	*p*	Estimates	CI	*p*
Group [combination]	0.39	− 2.69–3.47	0.802	1.41	− 1.32–4.15	0.307
Time [days]	− 0.13	− 0.17 to − 0.08	< 0.001***	− 0.05	− 0.09 to − 0.02	0.005**
Site [VA]	0.37	− 3.34–4.07	0.844	− 4.59	− 8.08 to − 1.11	0.011*
Group [combination] × time	− 0.02	− 0.08–0.04	0.443	0.02	− 0.03–0.07	0.359

Monotherapy: divalproex plus placebo; combination: combination therapy of divalproex plus blinded lithium and divalproex plus blinded quetiapine

VA Palo Alto

*p < 0.05, **p < 0.01, ***p < 0.001

### Longitudinal analyses

#### Young Mania Rating Scale

Results failed to demonstrate significant longitudinal differences in YMRS scores between the PBO and Li arms as well as between the PBO and QTP arms (see Table 5). A linear mixed model excluding the interaction term demonstrated a significant main effect of time in the entire cohort, (*b* = − 0.14, ß = − 0.43, *SE* = 0.01, CI [− 0.17, − 0.11], *t*(407) = − 9.86, *p* < 0.001).

**Table 5:** Longitudinal Outcomes in YMRS and HAM-D: monotherapy versus individual combination therapies

Predictors	YMRS			HAM-D		
	Estimates	CI	*p*	Estimates	CI	*p*
Group [Li]	1.80	− 1.77–5.37	0.319	2.29	− 0.87–5.45	0.153
Group [QTP]	− 0.83	− 4.36–2.69	0.640	0.66	− 2.46–3.78	0.675
Time [days]	− 0.13	− 0.18 to − 0.08	< 0.001***	− 0.05	− 0.09 to − 0.02	0.004**
Site [VA]	0.41	− 3.31–4.13	0.826	− 4.60	− 8.14 to − 1.07	0.011*
Group [Li] × time	− 0.06	− 0.14–0.01	0.108	− 0.00	− 0.06–0.05	0.878
Group [QTP] × time	0.00	− 0.06–0.07	0.919	0.04	− 0.01–0.09	0.124

PBO: monotherapy divalproex plus placebo [reference group]; Li: combination therapy of divalproex plus blinded lithium; QTP: divalproex plus blinded quetiapine; VA: Palo Alto VA

*p < 0.05, **p < 0.01, ***p < 0.001

#### Hamilton Depression Rating Scale

**Fig. 2:**
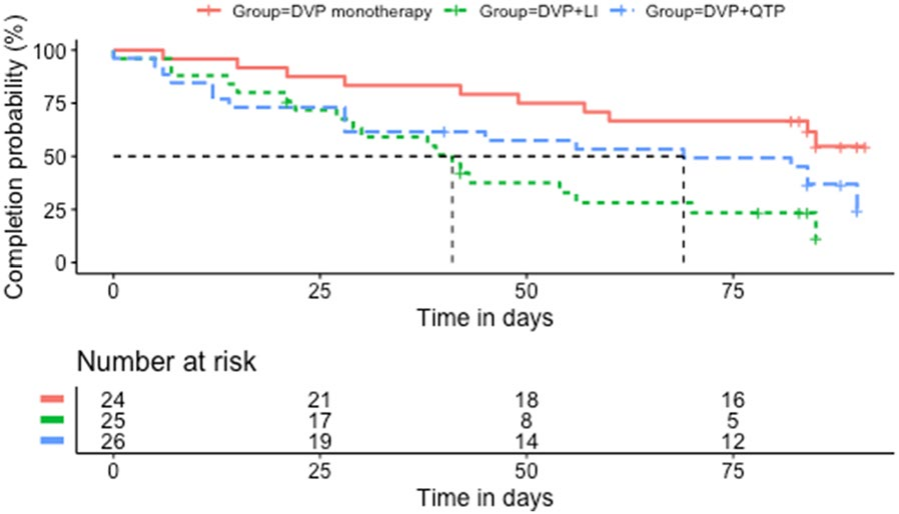
Kaplan–Meier time-to-event curves

Results failed to demonstrate significant longitudinal differences in HAM-D scores between the PBO and Li arms as well as between the PBO and QTP arms (see Table 5). On average, throughout the trial, HAM-D scores were lower at the Palo Alto VA (n = 6) than at UT Southwestern, (*b* = − 4.60, ß = − 4.62, *SE* = 1.77, CI [− 8.14, − 1.07], *t*(71) = − 2.60, *p* = 0.011) (see Table 5). A linear mixed model excluding the interaction term, demonstrated a main effect of time in the entire cohort, (*b* = − 0.04, ß = − 0.17, *SE* = 0.01, CI [− 0.06, − 0.02], *t*(407) = − 3.35, *p* = 0.001). See Fig. 2 for survival analyses.

### Time-to-dropout analyses

**Fig. 3:**
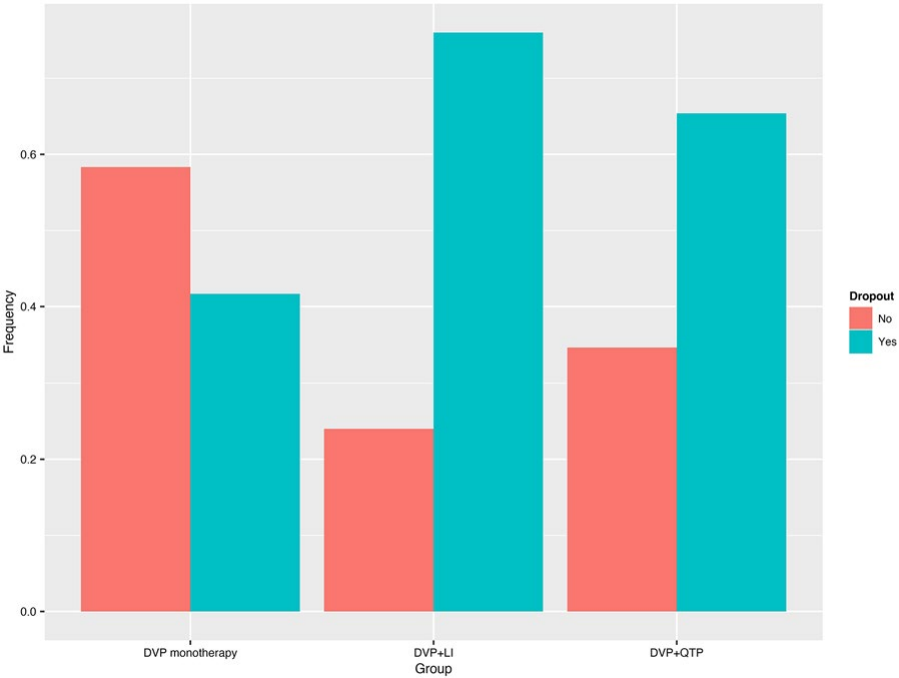
Frequency of dropout per study arm

The estimated median time-in-study for the Li and QTP groups were 41 and 69 days, respectively (see Fig. 3). The estimated median time-in-study was not reached for the PBO group. The Cox proportional hazards model failed to demonstrate a significant interaction between PBO versus Li and side effect severity as well as PBO versus QTP and side effect severity (see Table 6). Similarly, a separate model failed to find a significant interaction between PBO versus Li and number of side effects reported, (*b* = − 0.05, Wald = − 0.18, *p* = 0.859) as well as PBO versus QTP and number of side effects reported (*b* = − 0.00, Wald = − 0.00, *p* = 0.998).

**Table 6:** Cox regression on retention for monotherapy versus individual combination therapies

Variable	B	SE	Wald	*P*	Hazard ratio	95% CI
Group						
PBO	–	–	–	–	–	–
Li	1.11 (1.11)	0.53 (0.52)	2.11 (2.14)	0.035* (0.033)	3.04 (3.04)	1.08–8.57 (1.10–8.43)
QTP	0.27 (0.17)	0.58 (0.59)	0.46 (0.29)	0.644 (0.773)	1.31 (1.18)	0.42–4.06 (0.38–3.73)
Side effect	0.08 (0.09)	0.11 (0.11)	0.76 (0.78)	0.448 (.435)	1.09 (1.09)	0.88–1.35 (0.88–1.35)
Side effect severity × Li	− 0.05 (− 0.04)	0.12 (0.13)	− 0.43 (− 0.33)	0.666 (0.742)	0.95 (0.96)	0.75–1.21 (0.75–1.23)
Side effect severity × QTP	− 0.03 (− 0.03)	0.11 (0.11)	− 0.30 (− 0.29)	0.763 (0.769)	0.97 (0.97)	0.77–1.21 (0.78–1.21)
YMRS	0.00 (0.00)	0.03 (0.03)	0.11 (0.84)	0.914 (0.933)	1.00 (1.00)	0.95–1.06 (0.95–1.06)
HAM-D	0.06 (0.06)	0.04 (0.04)	1.64 (1.59)	0.101 (0.113)	1.06 (1.06)	0.99–1.14 (0.99–1.14)
Site	− 0.68	1.06	− 0.64	0.521	0.51	0.06–4.06

Estimates for the model that excluded site are shown in parentheses

PBO: monotherapy divalproex plus placebo; Li: combination therapy of divalproex plus blinded lithium; QTP: divalproex plus blinded quetiapine

“–” indicates reference group

*Significant at *p* < 0.05

The Cox regression estimated that the risk-of-dropout for a participant with an average side effect severity score was 3.04 times higher for the Li group than for the PBO group (see Table 6), albeit with a large confidence interval (95% CI = 1.08–8.57). The simple effect for the risk-of-dropout between the PBO and QTP groups was not significant (see Table 6 and Fig. 3). There were also no significant effects of YMRS and HAM-D or Site (see Table 6). Importantly, significance remained unchanged when we excluded the six participants who were recruited to the Palo Alto VA. Moreover, estimates were negligibly different across models (see Table 6). In the monotherapy and aggregated combination therapy model, the estimated risk-of-dropout was 2.56 times higher for the combination therapy arms than for the monotherapy arm (see Table 7). Although significance for HAM-D changed when excluding Palo Alto VA participants, the Hazard ratio was negligibly different (1.06 vs. 1.07) (see Table 7).

**Table 7:** Cox regression on retention for monotherapy versus aggregated combination therapies

Variable	B	SE	Wald	*P*	Hazard ratio	95% CI
Group						
Monotherapy	–	–	–	–	–	–
Combination therapy	0.93 (0.92)	0.48 (0.48)	1.94 (1.90)	0.052 (0.058)	2.56 (2.51)	0.99–6.61 (0.97–6.50)
Side effect severity	0.02 (0.02)	0.08 (0.09)	0.27 (0.27)	0.785 (0.785)	1.02 (1.02)	0.87–1.21 (0.86–1.21)
Side effect severity × group	0.01 (0.00)	0.09 (0.09)	− 0.43 (0.01)	0.939 (0.992)	1.01 (1.00)	0.85–1.20 (0.84–1.19)
YMRS	0.00 (− 0.00)	0.03 (0.03)	0.04 (− 0.03)	0.965 (0.973)	1.00 (1.00)	0.95–1.06 (0.95–1.05)
HAM-D	0.06 (0.07)	0.03 (0.03)	1.74 (2.06)	0.082 (0.039*)	1.06 (1.07)	0.99–1.14 (1.00–1.14)
Site	− 1.02	1.05	− 0.97	0.334	0.36	0.05–2.85

Estimates for the model that excluded site are shown in parentheses

Monotherapy: divalproex plus placebo; combination: combination therapy of divalproex plus blinded lithium and divalproex plus blinded quetiapine

Monotherapy (“–”) was used as the reference group

*Significant at *p* < 0.05

## Discussion

The primary hypothesis of this pilot trial was whether combination therapies (DVP + Li and DVP + QTP) would be associated with greater reduction in symptoms of hypomania and mania as measured by the YMRS than monotherapy alone (DVP + PBO), while the secondary hypothesis of this exploratory trial was whether combination therapies would be associated with lower depression levels as measured by the HAM-D than those on monotherapy. In lieu of the other original secondary measures of the present pilot study, a post-hoc exploratory aim was included to examine whether the effect of side effect severity on study retention differed among combination therapies and monotherapy (DVP + PBO) during the 12-week acute phase. Given the small sample size and high rates of attrition, the results of this pilot trial must be interpreted with caution and considered as exploratory. Contrary to the primary and secondary hypotheses, results failed to demonstrate significant longitudinal differences in mania and depression scores between combination therapies and monotherapy. Moreover, the effect of side effect severity on study retention did not vary as a function of monotherapy versus combination therapies. However, the risk-of-dropout was significantly greater in the DVP + Li arm versus the DVP + PBO arm.

Our provisional and exploratory null results for monotherapy versus combination therapies on symptoms of bipolar I disorder are consistent with prior literature for studies in ambulatory versus inpatient samples. For example, in a sample of 283 adult patients with bipolar I or II disorder, a prior study failed to find differences in clinical outcomes between lithium plus optimized personalized treatment (OPT) and OPT-only (Nierenberg et al. 2013). However, the OPT-only group was more frequently prescribed second-generation antipsychotics (Nierenberg et al. 2013). Similarly, a trial of lithium monotherapy, sertraline monotherapy or combination therapy of lithium plus sertraline in 142 patients with bipolar II disorder, reported no differences in treatment response or switch rate among groups (Altshuler et al. 2017). Consistent with our observation of increased rate-of-dropout in the DVP + Li arm versus the DVP + PBO arm, the study found an increased rate-of-dropout in the combination therapy arm than in the monotherapy arms (Altshuler et al. 2017). An open-label trial of lithium monotherapy, divalproex monotherapy or their combination for bipolar I relapse prevention in 330 participants found that combination therapy with lithium and divalproex was superior to monotherapy with divalproex but not superior to monotherapy with lithium for relapse prevention (Geddes et al. 2011). Taken together with findings from our pilot study, these data suggest an equivocal additional benefit of combination therapies (vs. monotherapy) for bipolar disorders.

Interestingly, while the rate-of-dropout differed between the DVP + Li and DVP + PBO arms, with a 3.04 times higher risk-of-dropout in the DVP + Li arm, the rate-of-dropout did not differ significantly between the DVP + QTP and DVP + PBO arms. It is essential to note that this null effect may be due to the limited number of participants per treatment arm and resulting low power. Indeed, an examination of Fig. 3 suggests a potential difference in rate-of-dropout, in the expected direction, between the DVP + QTP and DVP + PBO arms. Moreover, provisional findings from this pilot study did not suggest between-group differences in the effect of side-effect severity and number of side effects on rate-of-dropout, which may also be due to being underpowered. Further studies, with adequate numbers of participants to be fully powered, are needed to elucidate between-group differences in side effect severity on rate-of-dropout.

A consequential limitation of the present pilot study is the small sample size and differential attrition across treatment arms. The higher rate-of-dropout in the DVP + Li arm may have also violated the missing at random assumption of linear mixed models and increased Type II error in a potentially significant fashion. Future studies would benefit by the use of larger sample sizes to estimate a pattern mixture model or a joint model of the longitudinal outcome process and the time-to-dropout process, thereby adjusting for the effect of dropout in estimating longitudinal differences in YMRS and HAM-D among the 3 treatment arms. In addition, the generalizability of our results may be tempered by the low to moderate YMRS cutoff score of ≥ 15. Further, future study designs could consider potential differential response patterns between individuals with syndromal hypomania vs. mania.

## Conclusions

Three provisional implications, cautioned by the small sample size of this pilot study, may be drawn from the present double-blind, placebo-controlled ambulatory pilot trial. First, the preliminary findings suggest no added benefit of combination therapy versus monotherapy with divalproex sodium for manic symptoms in ambulatory subjects with bipolar I disorder; second, divalproex plus blinded lithium could be associated with greater risk of dropout than divalproex alone; and third, study retention in our pilot investigation did not appear to be associated with differences in side effect severity or the number of side effects reported between treatment arms, with attrition and low statistical power critical contributors.

## Data Availability

The datasets used and/or analyzed during the current study are available from the corresponding author on reasonable request.

## Abbreviations

DVP: Divalproex
PBO: Placebo
Li: Lithium
QTP: Quetiapine
BDI: Bipolar I disorder
SCID: Structured Clinical Interview for DSM-IV
YMRS: Young Mania Rating Scale
HAM-D: Hamilton Rating Scale for Depression
CGI-BP: Clinical Global Impression Scale for Bipolar Disorder
SOFAS: Social and Occupational Functioning Scale
